# The Patient Role in a Federal National-Scale Health Information Exchange

**DOI:** 10.2196/41750

**Published:** 2022-11-04

**Authors:** Joshua C Mandel, J P Pollak, Kenneth D Mandl

**Affiliations:** 1 Microsoft Healthcare Redmond, WA United States; 2 Department of Biomedical Informatics Harvard Medical School Boston, MA United States; 3 Cornell Tech New York, NY United States; 4 Computational Health Information Program Boston Children's Hospital Boston, MA United States

**Keywords:** health information exchange, patient control, Health Insurance Portability and Accountability Act, HIPAA, patient record, information exchange, information sharing, health record, privacy, security, public health, health policy, health information, federal trusted exchange, insurance company, patient data

## Abstract

The federal Trusted Exchange Framework and Common Agreement (TEFCA) aims to reduce fragmentation of patient records by expanding query-based health information exchange with nationwide connectivity for diverse purposes. TEFCA provides a common agreement and security framework allowing clinicians, and possibly insurance company staff, public health officials, and other authorized users, to query for health information about hundreds of millions of patients. TEFCA presents an opportunity to weave information exchange into the fabric of our national health information economy. We define 3 principles to promote patient autonomy and control within TEFCA: (1) patients can query for data about themselves, (2) patients can know when their data are queried and shared, and (3) patients can configure what is shared about them. We believe TEFCA should address these principles by the time it launches. While health information exchange already occurs on a large scale today, the launch of TEFCA introduces a major, new, and cohesive component of 21st-century US health care information infrastructure. We strongly advocate for a substantive role for the patient in TEFCA, one that will be a model for other systems and policies.

## Introduction

Since medical records are usually stored where they are produced, when patients traverse sites of care their information often becomes fragmented. The 21st Century Cures Act called for a Trusted Exchange Framework and Common Agreement (TEFCA) to enable turnkey access to medical histories across organizations. TEFCA, which is expected to be implemented starting in 2023, provides a framework for participating organizations to exchange patient data and also anticipates patients retrieving their own records.

After an overview of TEFCA and the history leading to it, we define 3 principles to promote patient autonomy and control as “rules of the road” for national-scale health information exchange (HIE).

## Health Information Exchange: Past and Present

Query-based exchanges emerged in the 1990s. Successful examples, such as the Indianapolis Network for Patient Care and Research [1], led to community health information networks [2], then to regional health information organizations, later renamed HIEs.

Query-based exchanges have primarily supported treatment, and most were deployed in limited contexts or vendor-defined boundaries [3]. Though myriad exchange organizations [4] have proved financially unsustainable [5], many today are sustainable, such as the government-supported Massachusetts Health Information Highway and the nonprofit Manifest Medex. Carequality, a membership-based nonprofit, underpins exchange among HIEs, electronic health record (EHR) vendors, and others. Epic Systems, leveraging extensive market share, enables its customers to participate in exchange. The Commonwell Health Alliance manages HIE for non-Epic members. Prior to TEFCA, common agreements for cross-organizational data exchange have been proposed within individual networks, by the Carequality Interoperability Framework [6] and the Markle Foundation [7].

## Trusted Exchange Framework and Common Agreement

Entities, including HIEs, will apply to become Qualified Health Information Networks (QHINs), committing to standardize a technical framework and implement the Common Agreement.

The number of accessible patient records is anticipated to grow to over 200 million with nationwide reach [5]. TEFCA expands the purposes of use; information may be exchanged for treatment, operations, payment, public health, government benefits determination, and individual access.

Importantly, TEFCA expands the number of users authorized to query. Authorization is handled within a *hierarchical trust model*; a small number (~10) of QHINs will offer connectivity for on the order of 10,000 organizational participants including EHR vendors and health systems. These, in turn, authorize many users, likely on the order of millions of clinicians, insurance company staff, public health officials, and others under the current proposals. Responses to queries would be obligatory in the context of treatment or individual access, and permitted in other cases, except where prohibited by applicable laws [8]. Responsibilities for security enforcement are delegated. For example, a QHIN would trust a hospital to provision accounts and maintain credentials for its authorized users. In turn, the hospital would trust its users to comply with policies and laws.

## General Challenges for TEFCA and Information Exchange

As is evident in public comments [9], TEFCA’s goal of broad access brings challenges around privacy, security, and autonomy. As there is no consistent approach to verifying patient identity in health care, matching is probabilistic, based on demographics. Each query result might represent a “true positive” (correctly returning data), “true negative” (correctly returning no data), “false positive” (returning data from the wrong patient ), or “false negative” (not returning existing data). Systems to verify user identity and match records across care sites can be expensive and raise privacy concerns because they sometimes aggregate large amounts of identifiable data, including biometrics.

Though queries are audited, any authorized user may look up data about any patient with an expectation of automated, immediate responses. One organization’s security lapse or a user’s compromised credentials could allow a malicious actor to find information about any patient, a risk that grows as networks expand.

## Principles for Designing the Patient Role

### Overview

Because sharing protected health information for treatment is exempt from HIPAA’s (Health Insurance Portability and Accountability Act) requirement for patient-facing accounting of disclosures, patients have little visibility into when or with whom their health data are shared through HIE. A health care provider sharing a patient’s information need not obtain consent. HIPAA does not compel a provider to heed patient requests to restrict sharing. Notifying patients during front-desk registration through signed “consent for treatment” yields patients no opportunity to negotiate. Patient concerns about privacy breaches and misuse of their information in exchanges have been well documented [10,11].

As implementers of TEFCA continue to address challenges through technical and business controls leading up to the network’s launch, we propose 3 principles for meeting core objectives while recognizing and supporting patient autonomy and control, even if only a subset of patients are unsatisfied with default sharing permissions.

### Principle 1. Patients Can Query for Data About Themselves

Today, patients face substantial challenges in assembling their records across sites of care [12,13], and as a result, uptake of individual access to patient records has been slow [14]. Exercising “individual access” using query-based exchange under TEFCA affords transparency about what records exist and allows patients to determine where their records are stored, identify errors [15], and correct missing information from failed matching. A single point of access to one’s entire history of care may lead more individuals to seek out digital copies of their records [14].

To manage patient access, TEFCA anticipates that third-party “individual access providers” will verify a patient’s identity, execute queries, and share results with the patient. We prefer a design affording individual access as a first-class feature of all QHINs rather than adding the technical, security, and organizational complexity of third-party coordination. This design would enable QHINs to absorb the costs of patient identity verification, rather than outsourcing them to a new category of businesses that must establish revenue streams to offset these costs.

### Principle 2. Patients Can Know When Their Data Are Queried and Shared

The ability to see how one’s own data are being queried can serve as a check that the system is working as intended or as a leading indicator that something has gone wrong. Patients are well positioned to notice unexpected queries or to detect the absence of an expected query. Under TEFCA’s current policies, such details would be invisible to patients.

### Principle 3. Patients Can Configure What Is Shared About Them

It is not yet established whether the widespread availability of data for care always improves outcomes, and there may be unintended consequences. Research is needed to see whether unfettered access to prior opinions and diagnoses improves care and whether restricted access introduces risks or degrades care. Given concerns about insurability, legal consequences, and stigmatization, patients may even avoid care to prevent widely accessible documentation. Additionally, national-scale data availability raises concerns about access and disclosure by political, journalistic, or adversarial actors. The option to configure what is shared may help establish a new patient-doctor relationship if a soured prior relationship is apparent in the chart. During a diagnostic odyssey, sharing less may reduce second-opinion clinicians from becoming prejudiced by previous specialists’ assessments. Control over sharing may also help patients restrict queries about pregnancy-related care.

TEFCA currently does not provide control to patients. We recognize that permissive default settings that maximize access might satisfy a majority [16] and jumpstart network growth. We propose that patient concerns could be addressed pragmatically, starting with an all-or-nothing ability to opt out of exchange. More sophisticated controls could include (1) the ability to approve individual requests as queries are submitted and potentially (2) enabling access to a subset of encounters. For aspects of the record where a full picture is critical, purpose-built registries (eg, prescription drug–monitoring programs) provide accurate information irrespective of TEFCA.

If TEFCA-based exchange proves to become a data source for research and public health, patient autonomy to opt out of sharing may need to be balanced with requirements for unbiased data sets [17].

## Conclusions

Launching query exchange capability on a national scale is a vast and worthy undertaking. While details are in flux and there is a TEFCA roadmap for future improvements, we believe these principles enforcing patient rights to autonomy and control should be addressed in policy and technology from the initial TEFCA launch. This will increase the likelihood of programmatic success by preemptively addressing legitimate concerns by advocacy groups. Though HIE is widespread today, and generally without a well-defined and protected patient role, TEFCA could serve as a model to underpin a 21st-century, patient-centered health information economy.

## Abbreviations

EHR: electronic health record
HIE: health information exchange
HIPAA: Health Insurance Portability and Accountability Act
QHIN: Qualified Health Information Network
TEFCA: Trusted Exchange Framework and Common Agreement
